# Factors Associated with ICU Admission and In-Hospital Mortality in a Decade of HIV/AIDS Admissions to a Tertiary Hospital at the Epicenter of the Brazilian Epidemic (2015–2024)

**DOI:** 10.21203/rs.3.rs-10553719/v1

**Published:** 2026-08-15

**Authors:** Maxwell Zywica, Christopher Justin Hernandez, Pedro M Fonseca, Andressa Noal, Alexandre Giovelli, Mary Catherine Cambou, Weston Maier, Eddy Segura, Ivana Rosângela Dos Santos Varella, Breno Santos, Marineide Gonçalves de Melo, Karin Nielsen-Saines

**Affiliations:** UCLA David Geffen School of Medicine; UCLA David Geffen School of Medicine; Hospital Nossa Senhora da Conceição; Hospital Nossa Senhora da Conceição; Hospital Nossa Senhora da Conceição; UCLA David Geffen School of Medicine; UCLA David Geffen School of Medicine; Universidad Científica del Sur; Hospital Nossa Senhora da Conceição; Hospital Nossa Senhora da Conceição; Hospital Nossa Senhora da Conceição; UCLA David Geffen School of Medicine

**Keywords:** South Brazil, HIV epidemic, hospitalizations due to HIV, new HIV diagnoses, advanced HIV disease

## Abstract

**Background:**

South Brazil experiences disproportionately high HIV/AIDS morbidity and mortality. To characterize the regional epidemic profile, we evaluated HIV hospitalizations over time.

**Methods:**

In a retrospective cohort of adults hospitalized with HIV between 2015–2024 to a tertiary institution in Porto Alegre, data extracted from institutional records was analyzed for hospitalization outcomes. Severe outcomes (SO) were defined as critical care admission or in-hospital mortality.

**Results:**

Among 5218 infectious disease hospitalizations, 3596 were due to HIV (69%), encompassing 2376 participants; 1555 (65%) met WHO stage III/IV criteria for advanced HIV disease (AHD). Most patients were white (65%), male (53%), urban (65%). Among AHD participants, 77% had not completed high school. From 2015 to 2024, median age of patients increased from 41 to 44 years (p < 0.001). Declines in new HIV diagnoses following admission (p = 0.017) and AHD admissions (p = 0.015) occurred over time; new HIV diagnoses in AHD patients remained unchanged (p = 0.107). AHD case fatality rate of 11% remained stable (p = 0.287). Tuberculosis was the most prevalent reason for admission (21%, n = 488) followed by bacterial pneumonia (14%, n = 312) in AHD patients. Bacterial pneumonia was leading cause of admission in non-AHD patients (12%, n = 151). OIs and nosocomial infections were implicated in over half of in-hospital mortalities. Factors associated with SO were sepsis, pneumocystis pneumonia, nosocomial infections, non-opportunistic malignancy, tuberculosis, new HIV diagnoses and/or CD4 < 50 cells/mm^3^.

**Conclusion:**

In south Brazil, late HIV diagnoses and barriers to care appear to drive mortality. Targeted efforts to diagnose and engage patients in care earlier are warranted to prevent severe outcomes.

## Introduction

Porto Alegre, the capital of Rio Grande do Sul (RS), experiences a disproportionately high burden of HIV/AIDS compared with other Brazilian state capitals. Recent epidemiologic data from the Brazilian Unified Health System (Sistema Único de Saúde, SUS) demonstrates that HIV incidence and HIV-related mortality rates in south Brazil consistently exceed national averages, rendering Porto Alegre the highest ranking on Brazil’s HIV/AIDS severity index.^[1, 2]^ Moreover, RS is currently experiencing a generalized epidemic with a distinctive landscape characterized by predominantly heterosexual transmission of HIV type C.^[3, 4]^ Interestingly, the southern region of Brazil departs from national demographic trends, hosting a predominantly white population and a more gender-balanced HIV epidemic.^[3, 5]^

Although Brazil’s approach to HIV treatment provides free access to antiretroviral therapy since 1996 and availability of antiretroviral pre-exposure prophylaxis (PrEP) since 2017, high rates of AIDS-related hospitalizations in Porto Alegre suggest the presence of significant gaps in the clinical cascade, particularly regarding late diagnosis and delayed treatment initiation.^[6–8]^ Such advanced-stage presentations most often derive from opportunistic infections that may necessitate intensive care unit (ICU) admissions, complex treatment plans, and lengthy stays, imposing substantial economic burden on the public health system compared to the cost-effective maintenance of outpatient viral suppression. ^[9, 10]^

In the context of persistently high AIDS incidence and mortality, which are driven by unique regional socioeconomic factors and a more gender-balanced transmission pattern than the rest of the country, a detailed epidemiologic characterization of people living with HIV in south Brazil is necessary. This will enable a better understanding of the present epidemic and guide appropriate outreach and effective treatment of this vulnerable population. Moreover, over the last decade, south Brazil has experienced detrimental changes to HIV health care policy, dramatic flooding events in 2023, and repercussions of the COVID-19 pandemic that have disrupted the HIV care cascade.^[11–13]^ The present study aims to better understand the burden of HIV and advanced HIV disease admissions at a referral hospital in Porto Alegre, RS from 2015–2024.

## Methods

### Study design

We conducted a retrospective cohort study using hospital records from a tertiary-level infectious diseases referral hospital in Porto Alegre, Rio Grande do Sul, Brazil. The study evaluated all adults hospitalized in the infectious diseases ward with HIV between January 1, 2015 and December 31, 2024. Eligible patients were defined by reactive HIV rapid test results with confirmatory documentation in medical records of a serologic diagnosis, HIV viral load results, or registration in the Brazilian database for antiretroviral medication tracking (Sistema de Controle Logístico de Medicamentos, SICLOM). Deidentified patient-level data were extracted from the Brazilian Unified Health System (Sistema Único de Saúde) databases and institutional records to characterize demographics, admission diagnoses, HIV-related clinical parameters, and hospitalization outcomes, including discharge, transfer, intensive care unit (ICU) admission, and in-hospital mortality. Patients with World Health Organization (WHO) stage III or IV HIV were classified as Advanced HIV Disease (AHD).^[14]^

The study was reviewed and approved by the Research Ethics Committee of the Conceição Hospital Group (Comitê de Ética em Pesquisa do Grupo Hospitalar Conceição, CEP-GHC) in accordance with the Declaration of Helsinki under protocol number 23–141, issued on September 12, 2023. As this was a retrospective analysis with no direct contact with participants and no identifying information, the requirement for informed consent was waived by the ethics committee.

### Statistical Analysis

Data was analyzed with simple statistics of Chi-squared for categorical variables and either Wilcoxon Rank-Sum or Kruskal-Wallace tests for continuous variables as appropriate. Severe outcomes (SO) were defined as any ICU admission or in-hospital mortality. Case fatality rates were calculated from in-hospital mortalities. Significance was established at α < 0.05. Statistical analysis and figure generation were performed using STATA (Version 15; StataCorp LLC, College Station, TX, USA).

Generalized estimating equation (GEE) models were used to identify associated factors of severe outcomes among participants hospitalized with and without AHD. To account for the dependency between observations, given repeat admissions among patients over the 10-year study period, we specified a longitudinal data structure clustered by unique patient identifiers. The model utilized Poisson distribution with a log link function and an exchangeable correlation structure to account for within-subject correlation. Robust standard errors were utilized to directly estimate the Prevalence Ratios (PR) and 95% confidence intervals per year.

Backwards model building with a hybrid approach was used for multivariable analysis. Adjusted generalized estimating equations, also with a Poisson distribution, log link function, and exchangeable correlation structure were used to identify predictors of SO in all participants with HIV, participants with AHD, and participants with non-AHD. Variables with documented impact on human health and social determinants of health (such as age, race, sex, education status) were de facto included in the model. All variables reaching significance at alpha = 0.20 in the univariate analysis were included in the multivariable analysis. Covariates that failed to reach significance (p > 0.05) in the multivariable analysis were culled from the model. This process was initially performed with the overall HIV positive cohort (N = 3596) and subsequently repeated for the AHD cohort (n = 2297) and non-AHD cohort (n = 1229). Results from the multivariable analysis were presented as Incidence Rate Ratios (IRRs) with corresponding 95% confidence intervals (CIs).

## Results

Among 5218 infectious disease hospitalizations, over a ten-year period between January 1, 2015 to December 31, 2024, 3596 admissions (69%) were due to HIV, encompassing 2376 participants. Patients were mainly white (65%), male (53%), and from the Porto Alegre metropolitan area, with most having less than a high school education. Patients of black/mixed race comprised 35% of the population (n = 823) (Table 1). HIV parameters highlighted a severely immunosuppressed population with 1555 patients (65%) meeting WHO stage III/IV criteria for advanced HIV disease (AHD) and 1,322 (56%) having a CD4 cell count below 200 cells/ mm3 (Table 1). New HIV diagnoses were made in 493 admitted patients (21%). Antiretroviral nonadherence was also high at 57% (n = 1068) among 1,859 patients with a prior HIV diagnosis and treatment initiation (Table 1).

Following 2020, with the onset of the COVID-19 pandemic, the number of HIV hospitalizations decreased by 31%. Significant decreases in the annual proportions of AHD (PR 0.98, 95% CI [0.97–0.99], p < 0.001) and new HIV diagnosis (PR 0.97, 95% CI [0.94–0.99], p = 0.018) were observed, although small (Fig. 1). Meanwhile, opportunistic infections and case fatality rates (CFR) remained stable (p = 0.594, p = 0.078 respectively). The mean CFR was high, being 9% overall and 11% for the AHD cohort (Table 1, Figs. 1 & 2). Despite these stable rates, median age increased from 41 in 2015 to 44 in 2024 (p < 0.001). Female admissions also increased significantly over time (p = 0.005), with women reaching 57% of all admissions and 61% of AHD admissions by 2022 (Fig. 1, Supplemental Fig. 1A) but remaining stable without AHD (Supplemental Fig. 1B). While women increasingly comprised overall admissions, new HIV diagnoses occurred mainly among men (Supplemental Fig. 1C).

Patients with and without AHD were stratified by occurrence of SO to compare hospital courses and admission diagnoses (Table 2A, 2B). All patients with SO were significantly older, regardless of AHD status (Table 2), although median age was higher in patients without AHD than patients with AHD (mean age 46 vs 42 p < 0.001, data not shown). Admissions with SO displayed significantly longer durations of hospitalization with 62% of AHD (p < 0.001) and 44% of non-AHD (p < 0.001) admissions exceeding 30 days (Table 2). Importantly, new HIV diagnosis occurred in 29% (n = 123) of patients with AHD and SO (p < 0.001, Table 2A).

Infection burden was worse in patients with SO regardless of AHD status. Regardless of AHD status, both groups displayed higher rates of nosocomial infections. Most common opportunistic infections (OI) included tuberculosis (TB) (21%, n = 488), Toxoplasma encephalitis (12%, n = 247), CMV (10%, n = 253), esophageal candidiasis (9%, n = 200), pneumocystis pneumonia (9%, n = 196), and cryptococcal meningitis (5%, n = 108) (Table 2A). Similar rates of syphilis or hepatitis C coinfection occurred between AHD and non-AHD admissions, but for non-AHD patients, syphilis was more common without SO (11% vs 4%, p = 0.027) (Table 2B). Bacterial pneumonia was the leading cause of admission for non-AHD patients (12%, n = 152) and second for AHD patients (14% n = 311) following TB. Bacterial pneumonia was the most common diagnosis among AHD patients with SO, followed by pneumocystis pneumonia, tuberculosis, and other opportunistic infection (Table 2A) whereas non-AHD patients with SO held diagnoses of sepsis, nosocomial or resistant infections, or non-opportunistic malignancy (Table 2B). Finally, the majority of in-hospital mortality was due to OIs in AHD patients and community acquired or nosocomial infections in non-AHD patients (Fig. 2A, 2B).

Multivariable regression models using generalized estimating equations were performed to identify associations with SO among all participants, AHD, and non-AHD cohorts (Fig. 3). In the total cohort, no demographic differences were predictors of SO (Fig. 3A). However, a new admission diagnosis of HIV, CD4 levels below 200 cells/ mm3—particularly less than 50 cells/mm3—bacterial pneumonia, sepsis, nosocomial infections, opportunistic infections due to tuberculosis or pneumocystis pneumonia, and non-opportunistic malignancy were associated with SO (Fig. 3, Supplemental Table 1). In contrast, esophageal candida appeared as a protective factor (Fig. 3A). Factors associated with SO remained significant among the AHD group (Fig. 3B). Among admissions of participants without AHD, infectious covariates including bacterial pneumonia, sepsis, and nosocomial infections, as well as malignancy were associated with SO. In this population, non-opportunistic malignancy was highly associated with SO (Fig. 3C).

## Discussion

Our findings showcase the profile and drivers of SO among hospitalized patients living with HIV at a tertiary institution in south Brazil over a decade. Despite a decrease in number of hospitalizations following the COVID-19 pandemic, the composition of the patient population shifted to a higher proportion of women, particularly women with advanced disease, and an overall older population. Black/mixed people with HIV were overrepresented among hospitalized patients with HIV, considering that in this geographic area, the vast majority of the population is white.^[5]^ HIV case fatality rates, new HIV diagnosis during admission, and rates of AHD among hospitalized patients showed little to no decrease over ten years. Among hospitalized patients in south Brazil, the largest OI burden derived from tuberculosis while the frequency of OIs remained stable over the years. Severe outcomes were associated with lower CD4 cell counts, a new HIV diagnosis following admission, and the presence of concurrent OIs, bacterial pneumonia, or non-opportunistic malignancy.

The COVID-19 pandemic caused ubiquitous disruptions in the HIV care cascade, decreasing testing, prevention, treatment, follow-up appointments, and hospital admissions.^[13, 15–19]^ Notably, as hospital systems adjusted to contain the influx of COVID-19 cases, south Brazil experienced the largest post-pandemic decrease in hospitalizations for noncommunicable diseases.^[20, 21]^ For HIV hospitalizations specifically, previous studies demonstrated a 28% reduction in HIV hospitalizations from 2020 to 2021 in south Brazil.^[22]^ Our findings align with this prior literature, yet we failed to observe correspondent decreases in the proportions of AHD admissions or new HIV diagnosis, reinforcing the enduring burden of HIV in the region (9–14).^[7, 8, 18, 19, 22]^ Although Brazil witnessed decreasing time trends in HIV/AIDS diagnoses following the 2013 test and treat initiative (i.e., people living with HIV tended to be diagnosed earlier in the course of their disease), these downtrends evaporated in south Brazil after the onset of the COVID-19 pandemic.^[22–24]^ Given the 2025 report of a generalized HIV epidemic in the Porto Alegre metropolitan region, these unchanging rates of AHD and new HIV diagnoses among hospitalized patients, as well as increasing rates of HIV diagnoses in hospitalized women and patients with a higher education level emphasize the universalizing burden of HIV/AIDS in south Brazil.^[3]^

Our analysis demonstrated that women with advanced disease drive this sex-based shift in total HIV admissions, whereas men continue to predominate among new HIV diagnoses on admission. The south region of Brazil displays a more gender-balanced epidemic and has the highest rate of HIV in pregnancy in the country. Together with the increasing prevalence of HIV among hospitalized women seen in our data, this study further highlights that the HIV epidemic in South Brazil is often driven by heterosexual transmission.^[1–4]^

Our findings demonstrate that women more often receive an earlier HIV diagnosis, prior to the need for hospitalization but nevertheless are less likely to maintain themselves engaged in care and treatment, as others have also demonstrated.^[25–27]^ Our data emphasizes the need for testing, treatment, and longitudinal retention strategies to capture both traditional and nontraditional populations at risk. Despite the availability of cost-free HIV treatment in Brazil for decades, and the availability of free HIV PrEP access since 2017, Brazil’s Single Unified Health System has attempted to mitigate the influence of treatment barriers.^[28, 29]^ In fact, recent calls to action have emphasized the role of prevention in controlling the Brazilian HIV/AIDS epidemic.^[6]^ Current outreach approaches, however, mainly cater to perceived at-risk communities such as gay men. While these historically at-risk groups remain important targets, our data demonstrates the magnitude of the HIV burden among women. This reality demands reappraisal of where HIV outreach is most necessary in south Brazil.^[3, 4, 27]^

We noted increasing age among hospitalized patients with HIV in south Brazil, which underscores the burden of chronic inflammation in longstanding HIV infection.^[30, 31]^ The lifetime malignancy risk is elevated among people living with HIV and remains high even with adequate antiretroviral adherence.^[32–35]^ The association between some malignancies and severe outcomes in patients hospitalized with HIV potentially highlights the emerging impact of long term HIV infection. Public health efforts should be undertaken to ensure proper cancer screening and steady follow-up in patients living with HIV to reduce hospitalization for an aging population with longstanding HIV infection.

While malignancy had the strongest associations with SO, OIs and nosocomial infections predominated as the main causes of admission, death, and predictors of SO, representing a persisting clinical challenge among hospitalized patients with HIV. The rates of OIs observed coincided with Brazilian epidemiological patterns at large.^[8]^ Tuberculosis, for example, continues to represent the leading infectious burden for people living with HIV, regardless of regional stratification. Interestingly, no form of cryptococcosis was associated with SO in our study, despite its frequent occurrence in this population and well-documented association with poor outcomes in national and regional literature.^[7, 8]^ Given the profound immunosuppression and OI burden observed, patients present not uncommonly with comorbid infections and complex clinical pictures, necessitating prolonged admission and subsequently developing nosocomial infections.^[36]^ Additionally, bacterial pneumonia, which was heavily associated with SO, creates a fertile substrate for superimposed infection.^[37–39]^ The increased infectious burden and associated risk underpins the importance of prompt diagnosis and treatment of associated infections and the outpatient need for HIV screening, treatment, and prevention efforts.

The retrospective study design poses limitations due to potentially missing or incomplete data within hospital records. Factors such as patient evasion, high disease acuity, or lack of patient reporting additional history yielded documentation differences which limited our ability to extract and consider substance use, housing status or sexuality information in our analysis. Moreover, although the study took place at a large referral institution in the capital city of the state of Rio Grande do Sul, this data represents a hospitalized population in the city of Porto Alegre and subsequently reflects only a portion of the regional HIV epidemic.

Nevertheless, despite these limitations, our study provides insight into factors associated with critical care admissions and mortality in patients hospitalized with HIV in south Brazil. Continued efforts are necessary to facilitate early detection of opportunistic infections and minimize hospital length of stay. In addition to increasing treatment adherence, retention in care, and PrEP access, future strategies must address the shifting demographic landscape to curtail the HIV epidemic in south Brazil.

## Conclusion

Demographics are shifting among hospitalized patients living with HIV in south Brazil. The marked association of malignancy with severe outcomes prompts the question of early cancer screening in this aging population. The sustained burden of OIs among patients with newly diagnosed HIV identified on admission suggests late HIV diagnosis and barriers to care drive mortality. Targeted efforts to improve early diagnosis and consistent engagement in care are warranted so rates of severe outcomes in hospitalized patients with HIV decline.

## Supplementary Material

Tables

Tables are available in the Supplementary Files section.

Supplementary Files

This is a list of supplementary files associated with this preprint. Click to download.

- AHDSupplementalTables.docx
- Tables.docx

## Figures and Tables

**Figure 1:**
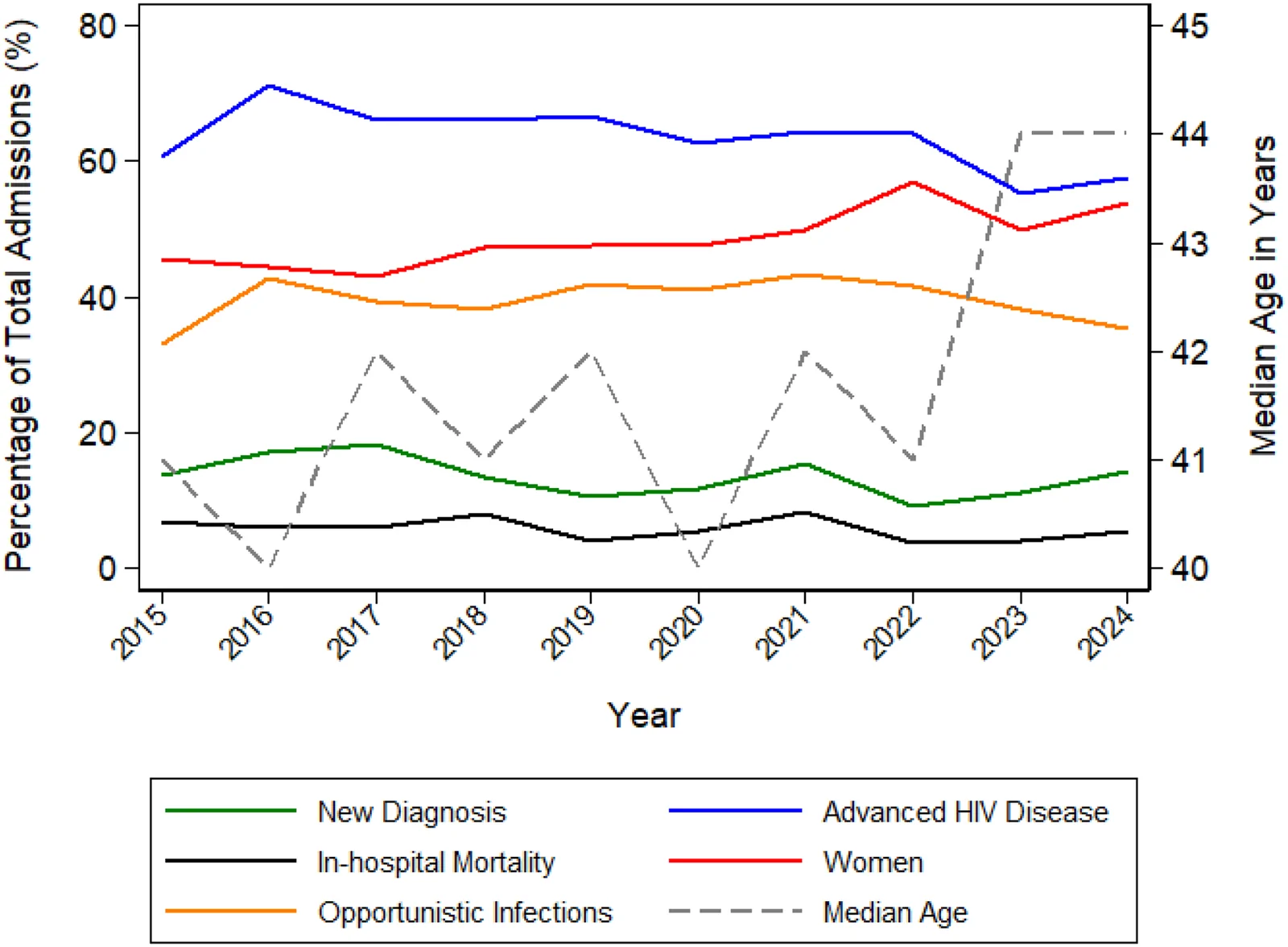
Time trends in composition of HIV admissions from 2015 – 2024. Line graph showing the annual proportions of admissions with advanced HIV disease, female sex, opportunistic infections, new HIV diagnosis, and in-hospital mortality for the right axis, and the median age coinciding with the left axis.

**Figure 2:**
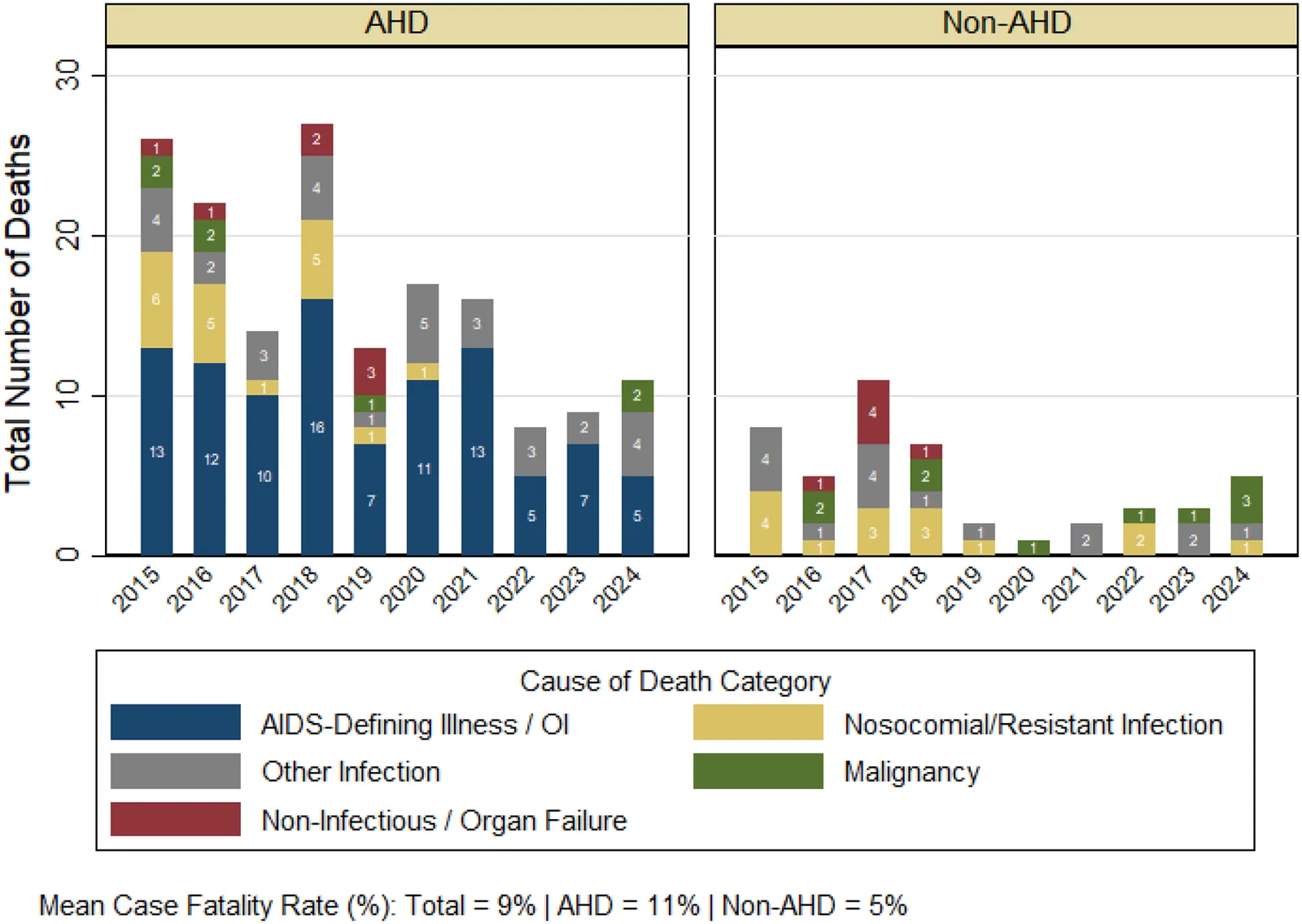
Causes of death and case fatality rate for patients with and without advanced HIV disease Bar graph showing the annual in-hospital mortalities and attributed cause of death for patients with and without advanced HIV disease and the case fatality rate for the total, AHD, non-AHD populations.

**Figure 3:**
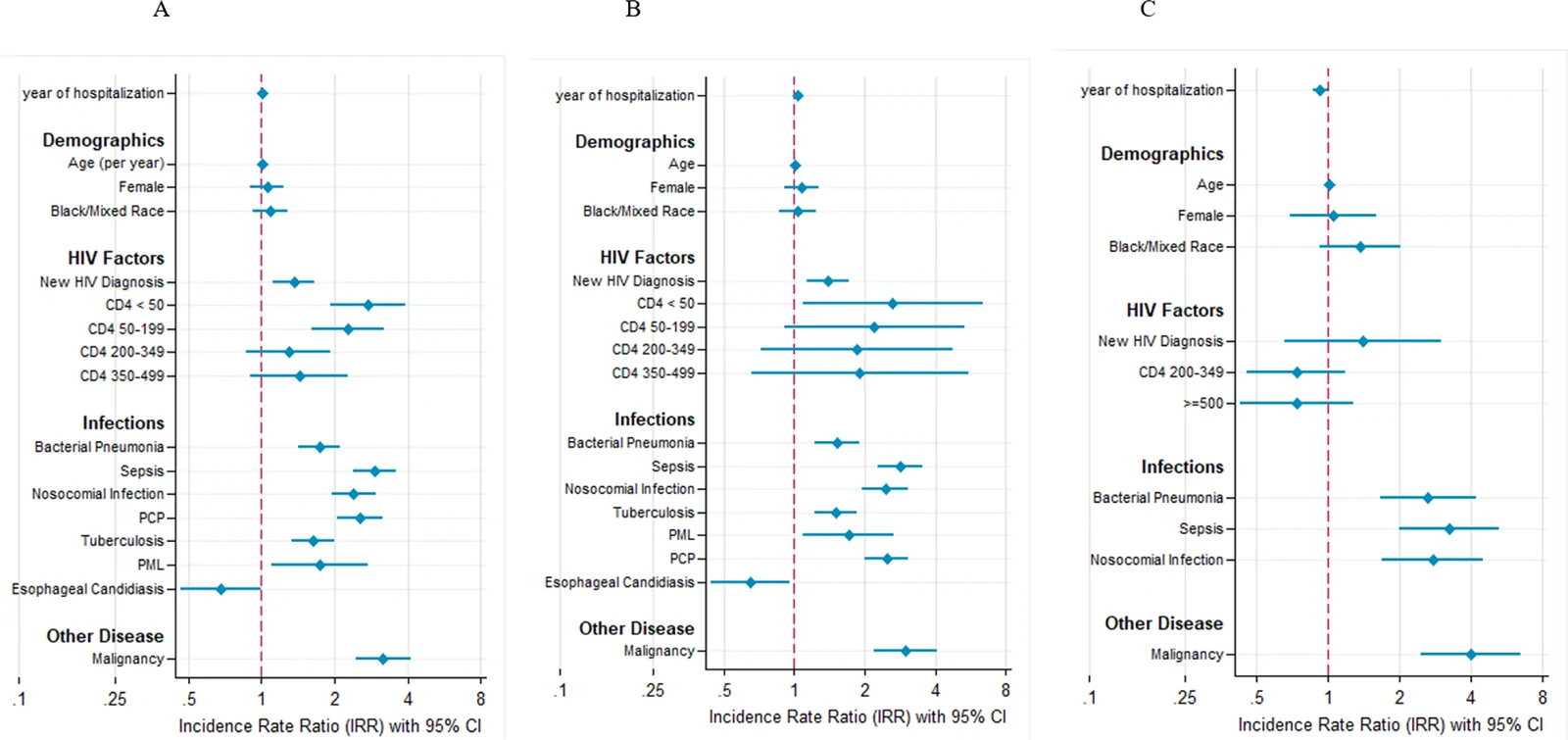
Forest Plots of associated factors with Severe Outcome for total population (A), AHD (B), and non-AHD (C) by multivariable regression with Generalized Estimating Equations Forest plots showing the adjusted incidence rate ratios (IRR) and 95% confidence intervals for (A) total population, (B) AHD patients, and (C) non-AHD patients.

## Abbreviations

AHD: Advanced HIV Disease (per WHO stage III/IV moderate to severe HIV disease criteria)
CEP-GHC: Comitê de Ética em Pesquisa do Grupo Hospitalar Conceição
CFR: Case Fatality Rate
CI: Confidence Interval
GEE: Generalized Estimating Equation
ICU: Intensive Care Unit
IRR: Incidence Rate Ratio
OI: Opportunistic Infection
PrEP: Pre-exposure Prophylaxis
RS: Rio Grande do Sul
SICLOM: Sistema de Controle Logístico de Medicamentos
SO: Severe outcome (critical care admission/ in-hospital mortality)
SUS: Sistema Único de Saúde (Brazilian Unified Health System
TB: Tuberculosis
WHO: World Health Organization
